# The Challenges of Predicting Rare Outcomes: A Critical Appraisal of Machine Learning Using the Pediatric Resuscitation and Trauma Outcome (PRESTO) Model in a Tanzanian Injury Registry

**DOI:** 10.1002/wjs.70553

**Published:** 2026-09-03

**Authors:** Joao Vitor Perez de Souza, Elizabeth M. Keating, William Nkenguye, Rosalia Njau, Happiness Kajoka, Pollyana Coelho Pessoa Santos, Edwin Joseph Shewiyo, Catherine A. Staton, Blandina T. Mmbaga, Joao Ricardo Nickenig Vissoci

**Affiliations:** ^1^ Duke Global Health Institute Duke University Durham North Carolina USA; ^2^ Department of Emergency Medicine Duke University School of Medicine Durham North Carolina USA; ^3^ GEMINI Research Center Duke Global Health Institute Duke University Durham North Carolina USA; ^4^ Division of Pediatric Emergency Medicine University of Utah Salt Lake City Utah USA; ^5^ Kilimanjaro Clinical Research Institute Moshi Tanzania; ^6^ Department of Epidemiology and Applied Biostatistics KCMC University Moshi Tanzania; ^7^ Pediatric Residency University of North Carolina UNC Chapel Hill North Carolina USA; ^8^ Department of Paediatric and Child Health KCMC University Moshi Tanzania

## Abstract

**Background:**

Injuries are responsible for 950,000 deaths per year among children and adolescents under 18 years old. Trauma prediction scores are useful in determining severity and prognosis of injury patients. The pediatric resuscitation and trauma outcome (PRESTO) score was developed as a simple score for short‐term mortality prediction in pediatric populations in low‐ and middle‐income countries (LMICs). Using variables available at the bedside in resource‐limited settings, PRESTO has been validated in South Africa, Rwanda, and Tanzania. In Tanzania, our team found these variables were present for most pediatric injury patients and that the model performed well in predicting mortality. However, we faced challenges due to small sample size and mortality being a rare outcome.

**Objective:**

We sought to determine whether we could improve predictability of the PRESTO model for pediatric patients by increasing sample size through inclusion of adult trauma patients from the same population at Kilimanjaro Christian Medical Center (KCMC) in Moshi, Tanzania.

**Design/Methods:**

Data were collected between November 2020 and February 2024 from a pediatric registry, and between April 2018 and February 2024 from an adult injury registry at KCMC. Missing data were addressed using multiple imputation. The dataset was split into training and testing sets (75/25). Ten machine learning algorithms were trained to predict in‐hospital mortality using clinical and demographic variables, with performance evaluated using cross‐validation, ROC‐AUC, sensitivity, and specificity.

**Results:**

A total of 5635 injury patients (911 pediatric and 4724 adults) were included. Pediatric in‐hospital mortality was 6.8%, while adult mortality was 4.4%. Pediatric patients presented with worse GCS scores compared to adults (4% severe vs. 1.4% severe). The best‐performing models were Random Forest (RF), C5, and Extreme Gradient Boosting (XGB), with ROC‐AUCs of 0.92, 0.90, and 0.81. In the test set, ROC‐AUC for pediatric patients was lower than for adults, with differences of 0.08, 0.06, and 0.05 for XGB, RF, and C5, respectively.

**Conclusions:**

Adding adult data did not improve predictability of the PRESTO model, possibly due to the fact that the lower mortality and injury severity in adults compared to children in our dataset statistically diluted the model. This supports the idea that children should be considered separately, especially for vital signs and mortality prediction. These findings highlight the challenge of predicting a rare outcome, and emphasize the need to increase pediatric registry sample sizes to develop more accurate models for mortality risk stratification in LMICs.

## Graphical abstract

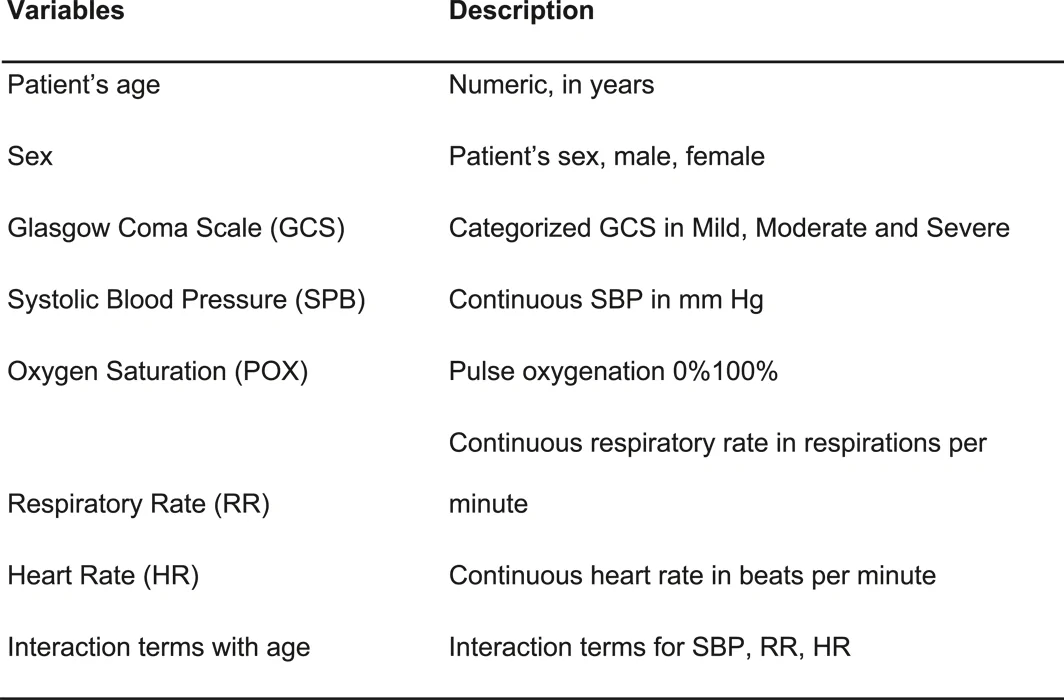

## 1 Introduction

Injuries are responsible for 950,000 deaths per year of children and adolescents under 18 years old worldwide, which includes 100 children dying per hour daily secondary to injuries [1]. Injury‐related events account for three of the top five causes of death for children and young adults aged 5–29 years [2]. The scenario is disproportionately worse in low‐and middle‐income countries (LMICs), which represents 95% of all injury‐related pediatric deaths worldwide [3]. Additionally, the majority of road traffic injuries, the leading cause of death in children aged 5–19 years old, happen in Sub‐Saharan Africa [3]. In Northern Tanzania, falls and road traffic injuries are the leading causes of injury in children less than 18 years old, while burns have the highest proportion of in‐hospital death [4, 5].

The use of trauma prediction scores has been shown to be helpful in determining the severity and prognosis of injury patients. This allows healthcare providers caring for these injured patients to determine the amount of resources and attention necessary in each case [6]. Although the pediatric trauma burden is disproportionately higher in LMICs, there are few trauma prediction models that are appropriate for use in LMICs [7]. Some have used adult models adapted to children or prediction scores originally validated in high‐income countries, but these fail to account for the different physiology of injured children and require information that is often not available in LMICs [8, 9]. To fill this gap, the pediatric resuscitation and trauma outcome (PRESTO) model was developed, presenting a feasible score of short‐term mortality prediction specific to pediatric populations in LMIC settings [10].

Using variables available at the bedside in resource‐limited settings, the PRESTO model has been validated in three LMICs including South Africa, Rwanda and, most recently, Tanzania [10, 11, 12, 13]. The PRESTO model includes heart rate, blood pressure, oxygen saturation, need for supplemental oxygen and the AVPU (alert, verbal, pain, unresponsive) scale of neurologic status assessment [10]. In Tanzania, our team found that these variables were present for the majority of pediatric injury patients and the model performed well in predicting mortality [13].

Despite the PRESTO model performing well in our population, our evaluations in Tanzania faced challenges with sample size when predicting mortality in pediatric injury patients, as it is luckily a rare outcome. Thus, in order to address our sample size challenge, in this study, we evaluate the accuracy of a machine‐learning model to predict child mortality with an extended sample that includes children and adult trauma patients from the same population at Kilimanjaro Christian Medical Center (KCMC) in Moshi, Tanzania. We aim to see if adding adults to increase our sample size increases our statistical power and predictive power of the PRESTO model. Improving our model would allow us to have an even better predictor of mortality to be used in future studies to better predict the outcomes of our pediatric injury patients and allocate resources accordingly.

## 2 Methods

### 2.1 Study Setting

The study was conducted at KCMC, a zonal referral hospital located in the Kilimanjaro region in Northern Tanzania, which serves a population of around 11 million people from both urban and rural areas [14]. The Emergency Medical Department (EMD) sees approximately 300 pediatric injury patients per year [4] and approximately 2000 adult injury patients per year [15].

### 2.2 Study Design and Population

This cross‐sectional study includes data from pediatric and adult prospective injury registries established at KCMC: (I) 911 pediatric injury patients seen in the emergency medical department (EMD) between November 2020 and February 2024 [4] for an injury sustained in the last month; (II) 4011 adult patients (i.e., 18 years of age or older) who sustained an injury in the last 24 hours, recruited between April 2018 and February 2024 [16]. Both registries are prospectively enrolling patients, including all pediatric and adult patients who present themselves at the KCMC EMD seeking care for acute injuries.

### 2.3 Ethical Approval

The pediatric injury registry was approved by the institutional review boards at the Tanzanian National Institute for Medical Research (NIMR/HQ/R.8a/Vol. IX/3475), Kilimanjaro Christian Medical Center (1252), and the University of Utah (IRB_00134560). The adult registry was approved by the institutional review boards at NIMR (NIMR/HQ/R.8c/Vol. I/1401), KCMC (497), and Duke University (Pro00062061). This investigation involves a de‐identified, secondary analysis of data sourced from these injury registries, and is focused on enhancing the quality of care for both adult and pediatric injury populations.

### 2.4 Data Collection

Trained Tanzanian research assistants were responsible for collecting patient information in the injury registries. They obtained data by directly observing patient care procedures and reviewing the medical record. For cases where patients arrived at the EMD outside the designated data collection periods, enrollment took place the following day if the patient remained hospitalized. However, patients who were discharged from the EMD during that time were not included. Throughout patients' hospital stays, the research assistants tracked and documented details of patient care and discharge information. All data were recorded using tablets via the Research Electronic Data Capture (REDCap) system [17, 18].

### 2.5 Study Variables

The outcome variable for this study was in‐hospital mortality. Independent variables selected for analysis included patient demographic characteristics, injury causation, and vital signs including systolic blood pressure (SBP), heart rate (HR), oxygen saturation, the need for supplemental oxygen (SO), and the patient's neurological status measured by the Glasgow Coma Scale (GCS) [19]. GCS scores were classified into severe [3, 4, 5, 6, 7, 8], moderate [9, 10, 11, 12, 13], and mild [14, 15]. These variables were used in calculation of the validated PRESTO model (Table 1) [13].

**TABLE 1:** Variables to calculate the pediatric resuscitation and trauma outcome (PRESTO) model.

Variables	Description
Patient's age	Numeric, in years
Sex	Patient's sex, male, female
Glasgow coma scale (GCS)	Categorized GCS in mild, moderate and severe
Systolic blood pressure (SPB)	Continuous SBP in mm Hg
Oxygen saturation (POX)	Pulse oxygenation 0%100%
Respiratory rate (RR)	Continuous respiratory rate in respirations per minute
Heart rate (HR)	Continuous heart rate in beats per minute
Interaction terms with age	Interaction terms for SBP, RR, HR

### 2.6 Data Analysis

#### 2.6.1 Data Preprocessing

Prior to model training, the data was preprocessed to address missing values and prepare the variables for analysis. Missing data was handled using multiple imputation with chained equations (MICE) [20]. MICE is a robust approach that leverages the relationships between variables to estimate plausible values for the missing data points. Fifteen versions of the dataset were generated using MICE and a representative version of the imputed data was used for downstream model training. The dataset was randomly split into training and testing sets using a 75/25 ratio while keeping the same proportion of pediatric and adult participants in the two sets. The training set was used to fit the machine learning (ML) models, while the testing set was reserved for final model evaluation. Numeric variables were standardized by centering at zero and scaling them to unit variance. Standardization is an important preprocessing step, as many ML algorithms perform better when the input features are on a similar scale [21]. Categorical variables were encoded using one‐hot encoding. This technique converts each categorical variable into a set of binary indicator variables, allowing the ML algorithms to better capture the information contained in the categorical predictors [22]. Differently from what was done by St‐Louis et al. [10], we expanded the predictor set by including age‐by‐vital‐sign interaction terms for HR, SBP, and RR. These terms were included because the clinical interpretation of vital signs may vary by age; for example, the same HR or RR value may reflect different levels of physiologic abnormality in younger versus older patients. The interaction terms therefore allowed the model to account for age‐dependent differences in the association between vital signs and the outcome.

#### 2.6.2 Machine Learning Models

A diverse set of 10 ML algorithms were evaluated for predicting patient outcomes: Extreme Gradient Boosting (XGB), Random Forest (RF), C5.0 Decision Trees (C5), Multilayer Perceptron, Logistic Regression, K‐Nearest Neighbors and Support Vector Machines with Radial Basis Function, Polynomial and Linear Kernels. To enable robust model evaluation, a 10‐fold cross‐validation procedure with 5 repeats was implemented. The training data was randomly partitioned into 10 equal‐sized folds, with the stratification on the outcome variable (death) to ensure a representative distribution of the target in each fold. Each fold was used once as a validation set, while the remaining 9 folds were used for model training. This process was repeated 5 times with different random data splits to obtain a more reliable estimate of the model's predictive performance. To optimize the performance of the ML models, a grid search approach of size 20 was used to tune the hyperparameters of each algorithm.

#### 2.6.3 Model Evaluation and Selection

The best hyperparameter settings for each model and the 3 best best‐ranked ML algorithms were determined based on the cross‐validation results based on their receiver operating characteristic area under the curve (ROC‐AUC). ROC‐AUC was chosen as the primary metric because it provides a comprehensive assessment of a model's ability to distinguish between positive and negative outcomes, regardless of the decision threshold [23]. The 3 best‐ranked models were then further evaluated on the held‐out testing set. The decision threshold was optimized to maximize model sensitivity while maintaining an acceptable level of specificity. This was done to ensure that the selected models would have a high ability to correctly identify positive cases (i.e., patients with the outcome of interest), which is particularly important in a clinical setting where missing true positives can have severe consequences.

*Reporting guideline:* this study was prepared following the *Transparent Reporting of a multivariable prediction model for Individual Prognosis Or Diagnosis (TRIPOD) guidelines* [24].

## 3 Results

A total of 4922 patients were included in our analysis, comprising 911 pediatric patients and 4011 adult patients from KCMC in Moshi, Tanzania (Table 2). The median age of the pediatric patients was 7.0 years (Interquartile Range [IQR]: 4.0–12.0), with 37% being female. The median age of the adult patients was 34 years (IQR: 26–47), with 18% being female. In‐hospital mortality was observed in 62 (6.8%) pediatric patients and 183 (4.4%) adult patients. Categorized causes of death for pediatric patients are shown in Table 3. Pediatric patients presented to the KCMC ED with lower Glasgow Coma Scale (GCS) scores compared to adult patients. Specifically, 7.2% of pediatric patients were in moderate condition compared to 5.1% of adults, and 4.0% of pediatric patients were in severe condition compared to 1.4% of adults.

**TABLE 2:** Distribution of variables included in the training of our machine learning model stratified by age group and in‐hospital mortality.

Variable	Pediatric			Adults		
	Overall, *N* = 911^a^	In‐hospital mortality		Overall, *N* = 4011^a^	In‐hospital mortality	
		No, *N* = 837^a^	Yes, *N* = 62^a^		No, *N* = 3,759^a^	Yes, *N* = 180^a^
Age (years)	7 (4, 12)	7 (4, 11.3)	6 (2.8, 12)	34 (26, 47)	33 (25, 46)	42 (28, 58)
Sex						
Female	332 (37%)	300 (36%)	25 (40%)	736 (18%)	694 (19%)	34 (19%)
Male	577 (63%)	535 (64%)	37 (60%)	3265 (82%)	3057 (81%)	144 (81%)
Missing	2	2	0	10	8	2
Systolic blood pressure (mmHg)	110 (100, 120)	110 (100, 120)	102 (94, 120)	124 (112, 135)	124 (112, 135)	121 (104, 137)
Missing	67	61	6	96	83	6
Oxygen saturation	98 (96, 99)	98 (96, 99)	95 (89, 98)	98 (96, 98)	98 (96, 99)	96 (94, 98)
Missing	36	33	3	110	96	4
Heart rate (BPM)	104 (89, 122)	103 (89, 121)	114 (82, 134)	85 (76, 97)	85 (76, 97)	86 (76, 107)
Missing	43	39	4	116	102	7
Respiratory rate (RPM)	24 (20, 28)	24 (20, 28)	26 (20, 30)	20 (19, 21)	20 (19, 21)	20 (19, 22)
Missing	89	80	9	577	511	40
Consciousness (GCS)						
Mild (GCS 14–15)	791 (89%)	748 (91%)	33 (57%)	3691 (92%)	3523 (94%)	102 (58%)
Moderate (GCS 9–13)	64 (7.2%)	53 (6.5%)	10 (17%)	202 (5.1%)	180 (4.8%)	22 (13%)
Severe (GCS 3–8)	36 (4.0%)	20 (2.4%)	15 (26%)	105 (2.6%)	53 (1.4%)	51 (29%)
Missing	20	16	4	13	3	5

Abbreviations: BPM, heart beats per minute; GCS, glasgow coma scale; RPM, respirations per minute.

^a^ Median (IQR); *n* (%).

**TABLE 3:** Categorized causes of death for pediatric patients in the cohort.

Categorized cause of death	*n*	%
Traumatic brain injury (TBI)/intracranial injury	23	37.1%
Burn injury (including burn + sepsis/respiratory failure)	18	29.0%
Cardiac/cardiorespiratory arrest (no clear underlying cause specified)	8	12.9%
Sepsis/septic shock (non‐burn primary)	4	6.5%
Abdominal trauma/acute abdomen	3	4.8%
Chest trauma	3	4.8%
Hemorrhagic shock/acute blood loss	1	1.6%
Anaphylaxis (bee sting)	1	1.6%
Other (multiorgan failure/DIC)	1	1.6%

The 3 best‐ranked ML models in predicting in‐hospital death were C5, RF, and XGB (Table 4). Cross‐validation results are presented in the Supporting Information S1: (File S1, Table S1 and Figure S1), while the specific hyperparameter configurations explored and their performance metrics are provided in the Supporting Information S2: File S2. During training, RF showed near‐perfect performance with an AUC of 1.00, while C5 and XGB had AUCs of 0.92 and 0.86, respectively. However, model performance decreased when applied to the test data, indicating some degree of overfitting. On the test set, all three models had similar AUC values, ranging from 0.73 to 0.76. Accuracy was modest at 66 to 67%, with sensitivity and specificity closely balanced between 0.65 and 0.67. This balance was achieved by adjusting the decision threshold to optimize both sensitivity and specificity, prioritizing overall model stability. Positive predictive values remained low, between 0.09 and 0.10, while negative predictive values were consistently high at 0.97. Models from the SVM family did not identify a clinically useful decision boundary at the default threshold. Although these models generated continuous prediction scores that allowed estimation of the AUC, their threshold‐based classification performance was inadequate, and they were therefore not considered suitable candidate models for clinical implementation. Full performance metrics, including raw outputs, threshold‐adjusted values, and confusion matrices for all ten models, are available in the Supporting Information S1: (Table S2−S3, Figures S2−S12, and Supporting Information S2: File S2).

**TABLE 4:** Performance metrics for the 3 best ranked models in the training and test sets after threshold optimization for the complete patient sample.

	Train						Test					
Model	AUC	Accuracy	Sensitivity	Specificity	PPV	NPV	AUC	Accuracy	Sensitivity	Specificity	PPV	NPV
C5	0.92	0.84	0.84	0.84	0.22	0.99	0.76	0.66	0.67	0.66	0.09	0.97
RF	1.00	0.99	0.99	0.99	0.80	1.00	0.73	0.66	0.65	0.66	0.09	0.97
XGB	0.86	0.78	0.78	0.78	0.16	0.99	0.76	0.67	0.67	0.67	0.10	0.97

*Note:* Sensitivity is also referred to as recall.

Abbreviations: C5, decision trees; NPV, negative predictive value; PPV, positive predictive value, also referred to as precision; RF, random forest; XGB, extreme gradient boosting.

When stratified by age group, model performance was consistently higher among pediatric patients compared to adults (Table 5). In children, all three models achieved higher AUC values, ranging from 0.79 to 0.84, whereas adult AUC values ranged from 0.71 to 0.74. Accuracy, sensitivity, and specificity were also more favorable in the pediatric subgroup, with values generally around 75 to 80%. Notably, the C5 model had the best performance in pediatric patients, with an AUC of 0.83 and sensitivity of 0.81. In contrast, adult model performance showed lower discrimination and classification metrics, with sensitivity around 0.62 across models and positive predictive values remaining below 0.10. These differences suggest that predictive models may perform more reliably in the pediatric population, possibly reflecting more distinct patterns of severity and presentation in younger patients.

**TABLE 5:** Performance metrics for the 3 best ranked models in these test sets after threshold optimization stratified by age groups.

	Adults (*n* = 1146)						Pediatric (*n* = 220)					
Model	AUC	Accuracy	Sensitivity	Specificity	PPV	NPV	AUC	Accuracy	Sensitivity	Specificity	PPV	NPV
C5	0.73	0.64	0.64	0.65	0.08	0.97	0.83	0.80	0.81	0.79	0.24	0.98
RF	0.71	0.63	0.62	0.63	0.07	0.97	0.79	0.75	0.75	0.75	0.19	0.97
XGB	0.74	0.65	0.62	0.65	0.08	0.97	0.84	0.75	0.75	0.75	0.19	0.97

*Note:* Sensitivity is also referred to as recall.

Abbreviations: C5, decision trees; NPV, negative predictive value; PPV, positive predictive value, also referred to as precision; RF, random forest; XGB, extreme gradient boosting.

## 4 Discussion

This study sought to respond to an important challenge in global pediatric emergency care: the lack of good quality and extensive data for pediatric injuries in LMICs to use to predict rare outcomes using machine learning methods. We tested the performance of the PRESTO model as a risk stratification algorithm using a combination of adult and pediatric patient data seen at the EMD at KCMC in Tanzania. Overall, we show that the addition of adult patients did not significantly increase model performance, despite a small but noticeable performance bump for pediatric patients. We also found that the variables needed to train a machine learning model like PRESTO were present in the majority of our patients, both pediatric and adult.

Despite representing a larger sample of our study population, adult patients showed lower injury severity overall. The pediatric patients in our registry had a slightly higher in‐hospital mortality rate and worse GCS scores on arrival to the KCMC EMD. This is in contrast to a study of blunt thoracic trauma from an injury registry in Durban, South Africa, that found that mortality in children was significantly lower in children than in adults [25]. It is possible that our findings are different due to the inclusion of all types of injuries and not just thoracic trauma. In contrast, our studies were similar to a study from a level I trauma registry in Houston, Texas, USA that demonstrated that in adults and children with pulmonary contusion, rates of death did not differ significantly between the groups [26]. It is possible that our findings are due to the fact that children have family caregivers that are able to bring them in for care even when they are more severely injured. It is also possible that our findings are due to the fact that our data is collected at a tertiary referral hospital and children who are less severely injured go to smaller, primary health facilities for care.

Previously, our team investigated the predictive performance of the PRESTO model in pediatric injury patients seen at the KCMC EMD, achieving an AUC for in‐hospital mortality of 0.81 [13]. Now, we demonstrate that adding adult injury patients to the sample of pediatrics did not improve the overall performance of our models (AUC 0.73–0.84). In fact, we report a higher predictive performance on the pediatric subset compared to adults, despite the much smaller sample representation in our dataset (Table 5). There are multiple explanations for this result. One explanation is that adult mortality is low in this registry and adults have a lower injury severity. Thus, adding adults to the model simply increased the “noise” of low‐acuity survivors. The model, already struggling to find the pediatric deaths, was now swamped by a larger volume of adult non‐events. This represents a statistical dilution effect. Another explanation of this result is the general idea that children are not just “little adults” and need to be considered separately, especially when it comes to vital signs and predicting mortality. Children differ anatomically and physiologically from adults, and often have different injury patterns and presentations [27, 28]. These differences have an impact on the management of pediatric injuries. Normal pediatric vital signs vary by age group, and are different from normal vital signs in adults [21, 22, 23, 27, 28]. Either way, we believe that increasing and diversifying the sample of local, setting‐specific pediatric injury patients is a necessity to develop and refine accurate and fair predictive models, especially in resource‐limited settings.

Despite the aforementioned clinical differences between adult and pediatric populations, the focus of this study is methodological rather than clinical. Given the limitations of a small pool of pediatric injury data, our initial goal was to improve our capacity to predict accurate outcomes in pediatric injury patients. Looking at evidence from broader artificial intelligence research, training on heterogeneous data can minimize diagnostic errors by increasing sensitivity and specificity, and thus improve reliability in actual clinical settings [29, 30]. So, to address the small pediatric sample size, we evaluated whether incorporating data from a large adult cohort could enhance our prediction performance. Unfortunately that is not the case, and we discovered that pediatric‐specialized data are necessary to achieve an accurate pediatric outcome prediction. Thus, bringing in more data does not necessarily equate to an improvement, as we need more precise data. This is supported by the literature, showing that overall specialized models are better than generalized models [31]. One study looking at modeling to predict acute appendicitis supports this conclusion and calls for separate analysis in subgroups such as pediatric patients to evaluate model performance separately [30].

In calibrating our model, we had to determine a threshold that balanced sensitivity and specificity that would adequately capture injured children who were at risk of in‐hospital mortality, while not over estimating those who would die. We leaned toward the side of over inclusion so that we would not miss a child who was at risk of dying. In calibrating the model coefficients for our population, we were able to derive a model that has good predictive potential. In trying to improve the PRESTO model, we calibrated the model coefficients for our population and attempted to derive a model that had even better predictive potential.

### 4.1 Limitations

This is a single‐site study which may limit the generalizability to other settings. In addition, we had a relatively small sample size of children with only 62 deaths. However, despite this our model performed well in predicting mortality in our previous study, and performed as in similar models published with larger sample sizes [5, 6]. Further, we may have survival bias in our population as patients in this study were well enough to survive to tertiary hospital evaluation, and sicker patients may have died before reaching care at KCMC. Thus, the true burden of injuries in Northern Tanzania has not yet been realized, and expanding the pediatric injury registry to include a pre‐tertiary hospital component is our next step in order to fully realize the extent of pediatric injuries in this area. In addition, the adult and the pediatric registries differed in their enrollment time frames, as patients in the adult registry had injuries within 24 hours and patients in the pediatric registry had injuries within the last month. This is due to the fact that the registries were set up at different times with slightly different goals. This study is a secondary analysis of the existing registries that were set up for quality improvement. In pediatrics we have found huge delays in reaching care, so we extended the registry to not miss children who are taking weeks to months to present to care in order to capture their data to try to find solutions to combat these delays. Despite the broader pediatric inclusion window, in‐hospital mortality was comparable across cohorts, with mortality of around 7.0% among pediatric patients and 4.5% among adult patients. Further, the broader inclusion window of the pediatric registry aligns with the existing literature validating the PRESTO model in Tanzania [13], as we used this very same registry to validate the PRESTO model in that study.

## 5 Conclusion

Adding adult data did not improve the predictability of the PRESTO model, possibly due to the fact that the lower mortality and injury severity in adults compared to children in our dataset statistically diluted the model. This supports the idea that children need to be considered separately, especially when it comes to vital signs and predicting mortality. These findings emphasize the importance of increasing the sample size of our pediatric injury registry to continue to collect locally relevant data to develop more accurate models for stratifying mortality risk. Such efforts will ensure that models are tailored to the unique needs of pediatric patients, particularly in LMICs, improving both predictions and resource allocation. Next steps include improving the model with more pediatric patients in pre‐tertiary hospital settings, and translating the PRESTO model into a clinically useful care process guideline to help guide treatment decisions in low‐resource settings.

## Author Contributions

**Joao Vitor Perez de Souza:** conceptualization, methodology, formal analysis, writing – original draft, writing – review and editing. **Elizabeth M. Keating:** conceptualization, supervision, writing – review and editing, writing – original draft, funding acquisition, data curation. **William Nkenguye:** writing – original draft, writing – review and editing, methodology. **Rosalia Njau:** writing – original draft, writing – review and editing. **Happiness Kajoka:** writing – original draft, writing – review and editing. **Pollyana Coelho Pessoa Santos:** writing – original draft, writing – review and editing, conceptualization. **Edwin Joseph Shewiyo:** writing – original draft, writing – review and editing. **Catherine A. Staton:** writing – original draft, writing – review and editing, supervision, investigation, conceptualization. **Blandina T. Mmbaga:** supervision, writing – review and editing, writing – original draft. **Joao Ricardo Nickenig Vissoci:** writing – original draft, conceptualization, investigation, methodology, data curation, supervision, formal analysis.

## Funding

This work was supported by the Fogarty International Center of the National Institutes of Health (D43 TW009337). EMK was supported in this work by the Eunice Kennedy Shriver National Institute of Child Health and Human Development (K23 HD112548). W.N., H.K. and E.J.S. were supported in this work by The Trauma Research Capacity Building Program at KCMC, funded by the D43 TRECK (D43‐TW012205).

## Conflicts of Interest

The authors declare no conflicts of interest.

## Supporting information


Supporting Information S1

Supporting Information S2

## Data Availability

The data that support the findings of this study are available on request from the corresponding author. The data are not publicly available due to privacy or ethical restrictions.
