# Supplementary material for: The Challenges of Predicting Rare Outcomes: A Critical Appraisal of Machine Learning Using the Pediatric Resuscitation and Trauma Outcome (PRESTO) Model in a Tanzanian Injury Registry
Source: World J Surg. 2026 Sep 3;50(10):3103–10. doi: 10.1002/wjs.70553 (PMC13662505; doi:10.1002/wjs.70553)
Supplement: Supplementary file 1 [file WJS-50-3103-s002.docx]

## Table S1. Cross Validation Results For the Best Instances of Each Model

| **Model** | **AUC** | **Accuracy** | **Sensitivity** | **Specificity** | **NPV** | **PPV** |
| --- | --- | --- | --- | --- | --- | --- |
| XGB | 0.813 ± 0.007 | 0.951 ± 0.001 | 0.059 ± 0.006 | 0.999 ± 0 | 0.952 ± 0.001 | 0.747 ± 0.045 |
| Random Forest | 0.812 ± 0.007 | 0.953 ± 0.001 | 0.133 ± 0.009 | 0.996 ± 0 | 0.956 ± 0.001 | 0.669 ± 0.039 |
| MLP | 0.805 ± 0.007 | 0.952 ± 0.001 | 0.1 ± 0.009 | 0.997 ± 0 | 0.954 ± 0.001 | 0.663 ± 0.045 |
| C5 | 0.801 ± 0.006 | 0.953 ± 0.001 | 0.124 ± 0.01 | 0.997 ± 0 | 0.955 ± 0.001 | 0.722 ± 0.039 |
| Logistic Regression | 0.786 ± 0.007 | 0.952 ± 0.001 | 0.145 ± 0.01 | 0.995 ± 0.001 | 0.956 ± 0.001 | 0.621 ± 0.036 |
| SVM RBF | 0.762 ± 0.009 | 0.949 ± 0.001 | 0 ± 0 | 1 ± 0 | 0.949 ± 0.001 | NaN ± NA |
| Naive Bayes | 0.761 ± 0.008 | 0.945 ± 0.001 | 0.138 ± 0.011 | 0.989 ± 0.001 | 0.956 ± 0.001 | 0.395 ± 0.027 |
| SVM Polynomial | 0.735 ± 0.009 | 0.949 ± 0.001 | 0 ± 0 | 1 ± 0 | 0.949 ± 0.001 | NaN ± NA |
| KNN | 0.725 ± 0.009 | 0.948 ± 0.001 | 0.168 ± 0.012 | 0.99 ± 0.001 | 0.957 ± 0.001 | 0.465 ± 0.029 |
| Decision Tree | 0.718 ± 0.009 | 0.943 ± 0.002 | 0.204 ± 0.014 | 0.983 ± 0.001 | 0.959 ± 0.001 | 0.384 ± 0.021 |
| SVM Linear | 0.627 ± 0.021 | 0.949 ± 0.001 | 0 ± 0 | 1 ± 0 | 0.949 ± 0.001 | NaN ± NA |

PPV, positive predictive value, also referred to as precision; NPV, negative predictive value; Sensitivity is also referred to as recall; RF, random forest;XGB, extreme gradient boosting; MLP, Multi-Layer Perceptron; SVM, Support Vector Machines; RBF, Radial Basis Function; KNN, K-Nearest Neighbors.

## Figure S1. Cross validation results for all model instances tested. PPV, positive predictive value, also referred to as precision; NPV, negative predictive value; Sensitivity is also referred to as recall; RF, random forest;XGB, extreme gradient boosting; MLP, Multi-Layer Perceptron; SVM, Support Vector Machines; RBF, Radial Basis Function; KNN, K-Nearest Neighbors.

## Table S2. Raw metrics for the 3 best-ranked models tested in this study

|  | Train | | | | | | Test | | | | | |
| --- | --- | --- | --- | --- | --- | --- | --- | --- | --- | --- | --- | --- |
| Model | AUC | Accuracy | Sensitivity | Specificity | PPV | NPV | AUC | Accuracy | Sensitivity | Specificity | PPV | NPV |
| C5 | 0.92 | 0.96 | 0.23 | 1 | 0.94 | 0.96 | 0.76 | 0.95 | 0.12 | 1 | 0.80 | 0.96 |
| Random Forest | 1.00 | 0.97 | 0.43 | 1 | 1.00 | 0.97 | 0.73 | 0.95 | 0.13 | 1 | 0.75 | 0.96 |
| XGB | 0.86 | 0.95 | 0.10 | 1 | 0.80 | 0.95 | 0.76 | 0.95 | 0.10 | 1 | 0.88 | 0.95 |

RF, random forest;XGB, extreme gradient boosting; MLP, Multi-Layer Perceptron; SVM, Support Vector Machines; RBF, Radial Basis Function; KNN, K-Nearest Neighbors.

## Table S3. Threshold-adjusted metrics for the models tested in this study

|  | **Train** | | | | | | **Test** | | | | | |
| --- | --- | --- | --- | --- | --- | --- | --- | --- | --- | --- | --- | --- |
| Model | AUC | Accuracy | Sensitivity | Specificity | PPV | NPV | AUC | Accuracy | Sensitivity | Specificity | PPV | NPV |
| C5 | 0.92 | 0.84 | 0.84 | 0.84 | 0.22 | 0.99 | 0.76 | 0.66 | 0.67 | 0.66 | 0.09 | 0.97 |
| XGB | 0.86 | 0.78 | 0.77 | 0.78 | 0.15 | 0.98 | 0.76 | 0.68 | 0.68 | 0.68 | 0.10 | 0.98 |
| Logistic Regression | 0.80 | 0.71 | 0.71 | 0.71 | 0.12 | 0.98 | 0.75 | 0.67 | 0.68 | 0.67 | 0.10 | 0.98 |
| SVM Poly* | 0.75 | 0.05 | 1.00 | 0.00 | 0.05 | - | 0.74 | 0.05 | 1.00 | 0.00 | 0.05 | - |
| MLP | 0.83 | 0.77 | 0.75 | 0.77 | 0.15 | 0.98 | 0.74 | 0.65 | 0.68 | 0.65 | 0.09 | 0.97 |
| Random Forest | 1.00 | 0.99 | 0.99 | 0.99 | 0.82 | 1.00 | 0.73 | 0.66 | 0.67 | 0.66 | 0.09 | 0.97 |
| SVM RBF* | 0.98 | 0.95 | 0.92 | 0.96 | 0.53 | 1.00 | 0.73 | 0.69 | 0.67 | 0.69 | 0.10 | 0.97 |
| KNN | 1.00 | 0.99 | 0.99 | 0.99 | 0.83 | 1.00 | 0.70 | 0.73 | 0.59 | 0.73 | 0.11 | 0.97 |
| Naive Bayes | 0.79 | 0.75 | 0.70 | 0.75 | 0.13 | 0.98 | 0.70 | 0.72 | 0.55 | 0.73 | 0.10 | 0.97 |
| SVM Linear* | 0.71 | 0.68 | 0.67 | 0.68 | 0.10 | 0.97 | 0.67 | 0.64 | 0.54 | 0.65 | 0.07 | 0.96 |
| Decision Tree | 0.76 | 0.89 | 0.57 | 0.90 | 0.24 | 0.98 | 0.67 | 0.87 | 0.46 | 0.89 | 0.18 | 0.97 |

PPV, positive predictive value, also referred to as precision; NPV, negative predictive value; Sensitivity is also referred to as recall; -, Not estimable; RF, random forest;XGB, extreme gradient boosting; MLP, Multi-Layer Perceptron; SVM, Support Vector Machines; RBF, Radial Basis Function; KNN, K-Nearest Neighbors.

For the SVM models, threshold adjustment produced unstable or clinically uninterpretable classification behavior. The polynomial SVM classified all observations as positive at the selected threshold, resulting in sensitivity of 1.00, specificity of 0.00, and an undefined NPV. The RBF and linear SVM models yielded valid threshold-adjusted classifications but showed substantial train-test performance differences, indicating limited generalizability.

## Table S4. Mechanism of Injury and Time from Injury to Arrival at KCMC in hours across the study sample.

|  | Pediatric | | | Adults | | | | |  | |
| --- | --- | --- | --- | --- | --- | --- | --- | --- | --- | --- |
|  |  | In-hospital mortality | |  | | In-hospital mortality | | |  | |
| Characteristic | N = 911^1^ | Yes   N = 62^1^ | No   N = 837^1^ | | N = 4,014^1^ | | Yes   N = 183^1^ | No   N = 3,806^1^ | | |
| Mechanism of Injury |  |  |  | |  | |  |  | | |
| Road Traffic Injury | 307 (34%) | 15 (24%) | 287 (34%) | | 2,551 (64%) | | 95 (53%) | 2,441 (64%) | | |
| Fall | 305 (34%) | 10 (16%) | 293 (35%) | | 723 (18%) | | 56 (31%) | 663 (17%) | | |
| Blunt Force/Struck/Hit | 142 (16%) | 9 (15%) | 132 (16%) | | 258 (6.4%) | | 9 (5.0%) | 248 (6.5%) | | |
| Assault | 0 (0%) | 0 (0%) | 0 (0%) | | 197 (4.9%) | | 4 (2.2%) | 190 (5.0%) | | |
| Burn | 105 (12%) | 25 (40%) | 76 (9.1%) | | 35 (0.9%) | | 12 (6.7%) | 22 (0.6%) | | |
| Penetrating Trauma | 15 (1.6%) | 1 (1.6%) | 14 (1.7%) | | 112 (2.8%) | | 0 (0%) | 112 (2.9%) | | |
| Other | 36 (4.0%) | 2 (3.2%) | 34 (4.1%) | | 131 (3.3%) | | 4 (2.2%) | 126 (3.3%) | | |
| Unknown | 1 | 0 | 1 | | 7 | | 3 | 4 | | |
| Injury to KCMC (hours) | 9 (3, 47) | 15 (4, 93) | 9 (3, 46) | | 6 (3, 15) | | 6 (3, 18) | 6 (3, 15) | | |
| Unknown | 10 | 5 | 5 | | 148 | | 15 | 132 | | |
| ^1^Median (Q1, Q3); n (%) | | | | | | | | | |  |

## Confusion Matrices: Raw vs Adjusted Thresholds

## Each figure shows the raw confusion matrix (threshold = 0.50) and the adjusted confusion matrix (balanced sensitivity/specificity threshold).

## Figure S2. Comparison of Raw and Threshold-Adjusted Classification for Logistic Regression

## Figure S3. Comparison of Raw and Threshold-Adjusted Classification for Random Forest

## Figure S4. Comparison of Raw and Threshold-Adjusted Classification for KNN

## Figure S5. Comparison of Raw and Threshold-Adjusted Classification for SVM Linear

## Figure S6. Comparison of Raw and Threshold-Adjusted Classification for C5

## Figure S7. Comparison of Raw and Threshold-Adjusted Classification for Naive Bayes

## Figure S8. Comparison of Raw and Threshold-Adjusted Classification for XGB

## Figure S9. Comparison of Raw and Threshold-Adjusted Classification for Decision Tree

## Figure S10. Comparison of Raw and Threshold-Adjusted Classification for SVM Poly

## Figure S11. Comparison of Raw and Threshold-Adjusted Classification for SVM RBF

## Figure S12. Comparison of Raw and Threshold-Adjusted Classification for MLP
